# Baseline fibroblast growth factor 23 predicts incident heart failure and cardiovascular mortality in patients with chronic kidney disease: A 3-year follow-up study

**DOI:** 10.1016/j.ijcha.2024.101587

**Published:** 2024-12-23

**Authors:** Ying Wang, Dingxin Zhang, Runzhe Zhou, Xiangjie Yang, Xiaoxia Wang, Yuxin Jiang, Xinyuan Zhou, Dashan Li, Jin Zhang, Yonggui Wu

**Affiliations:** a Department of Nephropathy, the First Affiliated Hospital of Anhui Medical University, Hefei, Anhui, People's Republic of China; b Department of Biostatistics of Epidemiology, School of Public Health, Anhui Medical University, Hefei, Anhui, People's Republic of China; c Cardiac Imaging Center, the First Affiliated Hospital of Anhui Medical University, Hefei, Anhui, People's Republic of China

**Keywords:** Fibroblast growth factor 23, Chronic kidney disease, Heart Failure, Preserved ejection fraction, α-Klotho, Cardiovascular mortality

## Abstract

**Background:**

Heart failure (HF) is a significant cause of death among patients with chronic kidney disease (CKD). Emerging data suggest a crucial role of fibroblast growth factor 23 (FGF23) in the pathogenesis of HF in CKD patients. The present study aimed to investigate whether the serum intact FGF23 (iFGF23) level is elevated when ejection fraction (EF) is preserved and to evaluate its predictive value for incident HF and cardiac mortality in CKD patients with preserved EF.

**Methods and results:**

We prospectively recruited 209 patients (mean age 52.7 ± 11.9 years, 37.3 % male) with CKD stages 3–5 and preserved EF, including those on peritoneal dialysis (PD) from a nephropathy center from November 2020 until July 2024.

**Results:**

Over a median follow-up of 29 (IQR 24–35) months, 60 (28.7 %) patients met the primary composite endpoints, including 53 (25.4 %) incident HF events and 7 (3.3 %) cardiac deaths. The cumulative incidence of composite endpoints was approximately 2-fold higher in patients with the highest quartile (Q4) level of lgiFGF23, compared with the lower quartiles (Q1-3). Baseline iFGF23 concentration was significantly associated with an increased risk of composite endpoint in the multivariable-adjusted Cox model, independent of kidney function, traditional cardiovascular risk factors, echocardiographic parameters, and α-Klotho. In a competing risk analysis, the Q4 level of lgiFGF23 (HR 2.43, 95 %CI 1.44–4.11; *P* = 0.001) was independently associated with HF and cardiac death.

**Conclusion:**

In CKD patients with preserved EF, serum iFGF23 was elevated before LVEF declined. A higher baseline serum iFGF23 level is significantly associated with the incidence of HF and cardiovascular mortality over a 3-year follow-up, demonstrating independent and incremental predictive value beyond traditional risk factors.

Cardiovascular complications remain the primary cause of death in patients with chronic kidney disease (CKD). Compared to the general population with normal renal function, the mortality risk of cardiovascular disease (CVD) in CKD patients is remarkably higher and increases even with a slight reduction in renal function or the presence of minor levels of albuminuria [1], [2]. Multiple factors contribute to the development of abnormal cardiac remodeling in individuals with declined renal function; however, the primary causes of cardiovascular deaths in individuals with advanced CKD are heart failure (HF) and sudden cardiac death [3]. The interrelation between kidney and cardiac dysfunction in CKD patients considered to be multifactorial and heterogeneous, is not fully comprehended. Emerging data suggest that the impact of traditional cardiac risk factors in CKD patients differs from that in the general population [2]. In fact, in advanced CKD patients and individuals undergoing dialysis, the common risk factors may not have a significant connection to an increased risk of mortality. In certain instances, there may even be an opposite relationship observed [2], [4], [5]. Meanwhile, the predictive power of traditional risk factors has been weakened as substantially increased CVD morbidity and mortality in CKD patients. In contrast, several uremia-related risk factors, for instance, inflammation, uremic toxins, oxidative stress, anemia, and disturbances of mineral metabolism, amplify traditional cardiovascular risk factors as renal function declines [6].

Fibroblast growth factor 23 (FGF23) is a hormone mainly secreted from osteocytes and osteoblasts and plays a vital role in regulating phosphate metabolism which has been found involved in the pathogenesis of cardiac alteration in CKD patients [7]. This regulation is maintained by coordinating how the kidneys manage phosphate, metabolize vitamin D, and release parathyroid hormone [8]. Following the removal of glycosylation and the signal peptide, FGF23 is released into the bloodstream [9]. FGF23 can be cleaved into inactive 18 kDa amino-terminal and approximately 12 kDa carboxy-terminal (cFGF23) fragments which can compete with full-length intact FGF23 (iFGF23) in binding to the FGF receptor (FGFR), therefore influencing certain biological processes [10]. iFGF23 excess may contribute to cardiac risks through direct induction of cardiac remodeling, vascular calcification, and renal sodium handling, or indirect decrease of calcitriol and α-klotho levels [11]. The α-klotho (abbreviated as klotho below) is a member of the Klotho proteins family and plays a role in facilitating the interaction between FGF and FGFR [12]. However, as neither cardiomyocytes nor cardiac fibroblasts express klotho [6], the role of klotho and its interaction with FGF23 in the heart is yet to be fully understood. There is a growing acknowledgment that FGF23 concentration elevates during the early stages of CKD and exhibits an exponential increase in correlation with the estimated glomerular filtration rate (eGFR). Elevated FGF23 is positively associated with the risk of cardiovascular diseases in the general population and patients with CKD including those on hemodialysis (HD) [13], [14]. This elevation may be through a klotho-independent manner in the heart to play a role in the etiology of structural and functional heart disease in patients with CKD [15]. In such a scenario, therapies targeting FGF23 might be therapeutically beneficial. However, few studies have measured both FGF23 and klotho levels simultaneously, and little is known about their roles in CKD patients with preserved ejection fraction (EF). In addition, the mechanisms linking FGF23 excess and klotho deficit in CKD patients undergoing peritoneal dialysis (PD) are less well-established.

We performed a prospective cohort study in patients with CKD stages 3–5 including those on PD and with preserved EF to investigate the degree of FGF23 level and its association with incident HF and cardiac deaths in such a CKD population. In addition, we explored whether the association was confounded by klotho concentration.

## 1 Methods

### 1.1 Study design and population

We prospectively recruited patients with CKD G3a-G5, A2–A3 of any cause [16] or receiving peritoneal dialysis (PD) admitted to our department from November 2020 to July 2024. The recruitment exclusion criteria were those who already have or had systematic HF based on the Framingham criteria [17], ischemic or hemorrhagic stroke, atherosclerotic CVD event, greater than moderate valve disease (defined as the presence of at least moderate stenosis or regurgitation of any heart valve determined by echocardiography), valve replacement, left ventricular ejection fraction (LVEF) < 50 %, peripheral arterial disease, acute inflammatory conditions such as peritonitis or autoimmune diseases. During the recruitment phase, a highly detailed and stepwise screening protocol was implemented. The initial evaluation involved a strict comparison of each potential individual's medical history, clinical parameters, and laboratory results with the pre-defined inclusion criteria. After this initial selection, only consented patients who met all the requirements would proceed to the next step of echocardiography examination. Based on the inclusion and exclusion criteria, a total of 211 individuals aged 18–78 years were recruited in this study for further follow-up for up to 3 years. All patients provided written informed consent. The study protocol was approved by the Clinical Research Ethics Committee of the First Affiliated Hospital of Anhui Medical University (Ethical review No. PJ2020-11-19).

### 1.2 Clinical features

Demographic characteristics, medical history, and medication history were ascertained through medical records and standardized questionnaires. Height and weight were measured with participants wearing light clothing and shoes removed. Body mass index (BMI) was calculated as weight (in kilograms) divided by height (in square meters). Waist circumference was measured by a tape measure at the halfway point between the lower costal border and the iliac crest. After resting for at least 5 min, supine systolic and diastolic blood pressure were tested twice and averaged for each patient. Hypertension was defined by averaged systolic/diastolic blood pressure ≥140/90 mm Hg or having antihypertensive medications. The diagnosis of diabetes was based on self-report including glucose-lowering treatment. Smoking history was categorized as ‘ever smoker’ including ‘past’ and ‘current’ smoker vs ‘never’ smoker.

### 1.3 Measurements

Peripheral venous blood samples (10 mL) from the cubital veins were collected on admission after overnight fasting of each participant. Blood samples (7 mL) and urine samples were sent to the clinical laboratory of the First Affiliated Hospital of Anhui Medical University for blood and urine routine tests to examine blood chemistry profiles and urine biochemical indicators. The results of all blood and urine routine tests and normal range were obtained through the electronic medical record system reported by the Department of Pathology. The eGFR was calculated using the Chronic Kidney Disease Epidemiology Collaboration (CKD-PEI) serum creatinine equation [18]. Subsequently, 3 mL blood samples were immediately separated in a refrigerated centrifuge and shipped on dry ice to the Center for Scientific Research of Anhui Medical University where investigators measured serum levels of full-length iFGF23 and α-klotho using enzyme-linked immunosorbent assay (ELISA) kit (Elabscience Biotech, Wuhan, CHN). The iFGF23 was measured in ng/ml, and α-klotho in pg/ml.

### 1.4 Six-minute walk test

The six-minute walk test (6MWT) was used to determine the exercise capacity of the participants. The testing walkway was an indoor and a hard, flat, 30-meter corridor. The maximum distance an individual could walk within a six-minute time frame was evaluated. Participants were allowed to set their own pace, and the test was carried out using a standardized protocol [19]. The six-minute walk distance (6MWD) was recorded.

### 1.5 Echocardiography

The GE Vivid E95 Cardiac Ultrasound was used for all echocardiography. In compliance with the guidelines of the American Society of Echocardiography [20], [21], each patient received a comprehensive echocardiogram that included standard transthoracic 2-dimensional, Doppler echocardiographic examinations, and speckle-tracking echocardiogram. The modified Simpson biplane method was utilized to measure and calculate LVEF. Left ventricular mass index (LVMi) was calculated from the M-mode LV mass measurement, and body surface area (BSA) was used to adjust it for body size. Diastolic indices including peak early diastolic velocities (E) and peak early diastolic mitral and lateral mitral annular velocity (e') as well as the ratio of mitral inflow early diastolic velocity to average e' velocity were obtained. For speckle-tracking imaging, three apical views (long-axis, 2-chamber, and 4-chamber views) were used to quantify longitudinal strain, which assessed the myocardial wall's shortening (negative strain) and lengthening (positive strain). Global longitudinal strain (GLS) was calculated by averaging three apical views using conventional software. In deformation analysis, shortening is expressed as a negative number; however, in this research, we express GLS without this information for computational simplicity (and since positive GLS were absent). Accordingly, diastolic dysfunction was defined as E/e’ >13.

### 1.6 Outcomes

During follow-up, regular telephone calls and questionnaires were used to identify potential symptoms of HF. Surveillance data were obtained through questionnaires and clinical visits, and medical records were requested for all-cause hospitalization and procedures. Patients with incomplete data were contacted through telephone interviews with their family members and excluded if data could not be obtained (2 patients). The diagnosis of incident HF was determined through a consensus process of three cardiologists reviewing all available information and verifying independently using the Framingham HF criteria [17]. LVEF was obtained in 47(94 %) cases with incident HF; the remaining 6 cases were diagnosed based on joint decision. The patient's nephrologist identified the reason for cardiac death, categorizing them as follows: presumed myocardial ischemia, myocardial ischemia/infarction, heart failure, stroke, and sudden cardiac death (which includes arrhythmia and cardiac arrest, with the cause being unclear whether it occurred in or out of the hospital); Seven cardiac deaths were identified and 17 non-cardiovascular deaths were recorded. The primary composite endpoint for the study was new-onset HF and cardiovascular mortality, and non-cardiovascular death was considered as a competing risk.

### 1.7 Statistical analysis

Statistical analyses were done with standard statistical computer software SPSS version 26 (IBM, Chicago, Illinois). Normally distributed variables were presented as mean ± standard deviation (SD), and non-normally distributed variables as medians with interquartile range (IQR). Categorical variables were presented as counts and percentages. Kolmogorov-Smirnov test was used to assess normality. Serum iFGF23 levels were not normally distributed, therefore log-10 transformed (lgiFGF23) to a normal distribution. The one-way ANOVA test was performed for comparison among different iFGF23 quartile groups. *P* < 0.05 was deemed to be statistically significant.

We first investigated the degree of iFGF23 elevation in the CKD population using iFGF23 quartiles. We used linear regression models to investigate the determinants of baseline serum iFGF23 concentration. We took the variables that satisfied P < 0.3 in the univariate linear analysis as candidates into multivariable regression analysis. Then we adopt the stepwise approach with thresholds of P < 0.05 as inclusion criteria and P > 0.10 as exclusion criteria to keep or remove variables respectively in the age- and gender-adjusted multivariable calculations. Longitudinal analyses that investigated the association of baseline iFGF23 concentration with the risk of new-onset HF and cardiovascular death, we performed Cox proportional hazards regression models. Baseline serum iFGF23 levels were expressed as a continuous variable with hazard ratios (HRs) and 95 % confidence interval (CI) calculated per standard deviation (SD) increment of lgiFGF23 and in quartiles. Covariates for adjustment were chosen based on previous associations as well-recognized prognosticators of HF and death (e.g. hypertension, and smoking), and potential confounders (e.g. kidney function, HF biomarker, echo parameters, and α-klotho level). Age and gender were treated as dummy variables in all models. Cox regression models were composed as follows: model 1, adjusted for age and gender; model 2, additionally adjusted for ever smoker, diabetes mellitus, hypertension, and peritoneal dialysis; model 3, as model 2 and additionally adjusted for hemoglobin, eGFR_CKD-Epi_, NT-proBNP, and α-klotho; model 4, as model 3 and additionally adjusted for E/e’ (fully adjusted model). In supplemental analyses, the bone mineral biomarkers (serum calcium, alkaline phosphatase, and PTH) and other echo parameters (LVMi, and GLS) as potential mediators were added separately into the fully adjusted model. Additionally, with the three lower iFGF23 quartiles 1–3 (Q1-3) defined as the reference group, HRs of the highest quartile (Q4) were calculated in the unadjusted and fully adjusted models. Then, the cumulative event-free survival rate was estimated and graphically displayed separately according to quartiles of the iFGF23 level by the Kaplan-Meier method, and log-rank testing was applied. To evaluate the incremental value of adding iFGF23 to conventional cardiovascular risk factors, Receiver-Operating Characteristic Curves (ROCs) were performed, and the area under the curves (AUCs) was calculated and compared.

### 1.8 Sensitivity analyses

Competing-risks regression was performed to adjust for non-cardiac mortality during follow-up as a competing risk in the survival analyses. As the highest HR and cumulative events of the primary endpoints have been identified in the highest iFGF23 quartile levels (Q4), cumulative incidence estimates of the primary endpoints were calculated and graphically displayed separately between CKD patients with Q1-3 and Q4 iFGF23 levels. Data were analyzed using Stata (version 18; StataCorp LP, College Station, Tx). P < 0.05 were considered statistically significant for all described analyses.

## 2 Results

### 2.1 Baseline descriptive characteristics

**Fig. 1:**
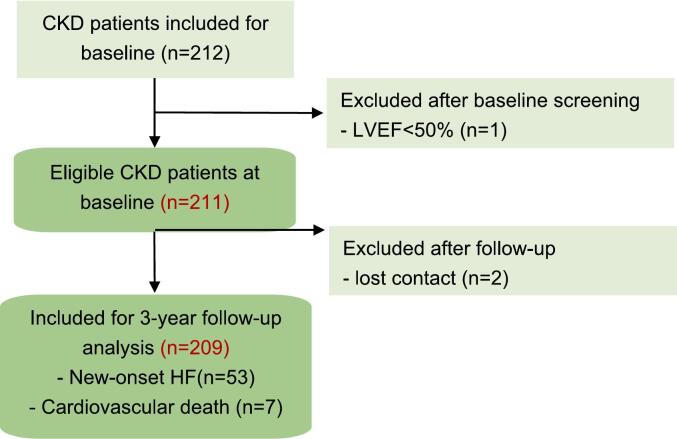
Flowchart of the original study group, reasons for exclusion, and patients enrolled in the study.

The overall study recruitment is shown in Fig. 1. After a median follow-up of 29(IQR 24–35) months, 2 (0.9 %) participants were lost to follow-up. During the follow-up, among the remaining 209 individuals, 60(28.7 %) met the primary endpoints including 53(25.4 %) incident HF and 7 (3.3 %) cardiovascular death. Among the remaining 149 patients, 13(6.2 %) had non-cardiovascular death. The baseline characteristics of the cohort and the baseline characteristics of the study population stratified according to the quartiles of iFGF23 are illustrated in Table 1. The mean age was 52.7 ± 11.9 years and 37.7 % were male. The median eGFR_CDK-EPI_ of the cohort was 5.76(3.64, 9.56) ml/min 1.73 m^2^, and more than half of the study population was on PD therapy (59.3 %). The median PD duration of 124 included PD patients was 26.50(IQR 9.25–55.5) months. All patients on PD were undergoing continuous ambulatory peritoneal dialysis (CAPD). The median serum iFGF23 was 157.97(IQR 80,53–321.16) ng/ml and the median α-Klotho was 1954.99(IQR 1002.20–3563.37) pg/ml. The mean 6MWD was (386.50 ± 87.92) meters. Compared to the no HF and cardiovascular death group, the mean lgiFGF23 value was significantly higher in the HF and cardiovascular death group (2.29 ± 0.46 vs 2.09 ± 0.44, *P* = 0.007).

**Table 1:** Baseline clinical characteristics, laboratory and echocardiographic parameters, and 6MWD according to quartiles of iFGF23 concentration.

**Variables**	**All (n = 209)**	**iFGF23 Quartile 1** **(<73.30 ng/ml)** **n = 52**	**iFGF23 Quartile 2** **(73.30**–**163.71 ng/ml)** **n = 52**	**iFGF23 Quartile 3** **(163.72**–**231.00 ng/ml)** **n = 52**	**iFGF23 Quartile 4** **(>231.00 ng/ml)** **n = 53**	*P* value*
iFGF23, ng/ml	157.97(80,53,321.16)	46.11(35.58,59.08)	131.47(99.26,155.91)	213.27(184.55,267.16)	483.47(328.00,898.48)	<0.001

**Demographics and clinical characteristics**						
Age(yrs)	52.74 ± 11.94	54.70 ± 12.48	52.60 ± 12.20	51.25 ± 13.334	52.94 ± 11.07	…#
Male gender, (%)	78(37.3)	22(42.3)	26(30.8)	17(32.7)	23(43.4)	…#
BMI, kg/m^2^	22.60 ± 3.44	22.38 ± 3.54	22.88 ± 3.34	22.91 ± 3.72	22.21 ± 3.20	0.954
WHR	0.95(0.90,0.99)	0.94(0.91,0.98)	0.95(0.90,0.99)	0.95(0.91,0.99)	0.94(0.90,1.00)	0.758
Heart rate, beats/min	81.45 ± 13.18	82.74 ± 13.11	78.10 ± 11.43	84.48 ± 13.60	80.46 ± 13.92	0.081
Systolic BP, mm Hg	145.66 ± 21.51	143.44 ± 21.92	148.81 ± 18.35	148.94 ± 23.32	143.44 ± 22.20	0.415
Diastolic BP, mm Hg	90.26 ± 12.74	89.53 ± 17.27	90.71 ± 8.94	92.44 ± 10.66	88.37 ± 12.44	0.761
Smoking status, ever (%)	43(20.6)	15(28.8)	10(19.2)	7(13.5)	11(20.8)	0.127
Hypertension, (%)	172(82.3)	40(76.9)	41(78.8)	46(88.5)	45（84.9）	0.269
Diabetes, (%)	36(17.2)	10(19.2)	8(15.4)	11(21.2)	7(13.2)	0.669
Peritoneal dialysis, (%)	124(59.3)	30(57.7)	31(59.6)	28(53.8)	35(66.0)	0.607

**Biochemical parameters (with normal range)**						
Hemoglobin (130 ∼ 175 g/L)	97.02 ± 22.21	98.66 ± 19.73	95.66 ± 22.09	97.96 ± 18.66	95.73 ± 27.81	0.413
MCV (82 ∼ 100 fl)	91.46 ± 5.14	91.29 ± 4.84	92.19 ± 5.12	91.68 ± 5.42	90.72 ± 5.21	0.723
Iron ( 7.8 ∼ 32.2 μmol/L)	11.93 ± 4.52	11.96 ± 4.32	11.73 ± 4.21	12.25 ± 4.86	11.99 ± 4.72	0.687
TSAT (33 ∼ 35 %)	27.41 ± 10.6	27.27 ± 9.02	28.3 ± 11.06	27.11 ± 10.85	28.04 ± 11.41	0.745
Ferritin (10 ∼ 120 μg/L)	219.00(101.00,338.00)	243.00(107.50,323.50)	181.00(114.00,369.00)	215.00(64.50,294.00)	226.50(106.50,416.50)	0.776
CRP (0 ∼ 10 mg/L)	2.30(0.78,6.64)	2.5(0.88,6.72)	1.40(0.54,5.20)	2.23(0.63,8.65)	2.65(0.73,11.75)	0.466
Urea nitrogen (8 ∼ 22 mg/dL)	54.79 ± 19.75	55.01 ± 21.12	54.79 ± 28.52	57.48 ± 21.10	51.74 ± 18.24	0.800
Creatinine (0.6 ∼ 1.1 mg/dL)	8.49 ± 4.34	8.20 ± 4.12	8.094 ± 4.08	8.35 ± 4.44	9.32 ± 4.70	0.180
eGFR_CKD-EPI_ (>90 ml/min per 1.73 m^2^)	5.76(3.64,9.56)	5.93(4.00,14.00)	6.61(3.52,10.64)	5.48(3.77,9.40)	4.57(3.51,7.48)	0.452
Serum albumin (40 ∼ 55 g/L)	36.23 ± 4.79	36.36 ± 4.49	37.09 ± 5.58	36.00 ± 4.38	35.85 ± 5.46	0.513
Total cholesterol (0 ∼ 5.17 mg/dL)	4.38(3.77,5.49)	4.08(3.67,5.08)	4.61(4.11,5.81)	4.27(3.78,5.55)	4.41(3.47,5.46)	0.656
Calcium (2.11 ∼ 2.52 mmol/L)	2.23 ± 0.24	2.20 ± 0.19	2.22 ± 0.18	2.21 ± 0.27	2.25 ± 0.26	0.216
Alkaline phosphatase (35 ∼ 100 U/L)	85.00(63.00,105.00)	102.00(78.50,121.00)	79.00(55.50,95.00)	87.00(66.00,100.0)	83.00(55.00,115.00)	0.799
PTH (15 ∼ 65 pg/ml)	176.00(80.25,354.00)	186.00(87.50,387.00)	165.00(70.00,352.00)	200.50(105.75,365.00)	158.00(64.00,326.00)	0.474
NT-proBNP (237.25 ∼ 432.75 pg/ml)	388.92 ± 111.67	139.25 ± 155.36	104.87 ± 131.65	255.85 ± 771.37	214.37 ± 319.37	0.112
CK-MB (0 ∼ 24 U/L)	12.00(9.00,15.00)	12.00(10.00,16.00)	11.00(9.00,15.50)	11.00(10.00,14.00)	8.50(6.75,13.25)	0.720
Serum phosphorus (0.85 ∼ 1.51 mmol/L)	1.66 ± 0.46	1.67 ± 0.40	1.59 ± 0.41	1.68 ± 0.46	1.70 ± 0.56	0.622
α-Klotho, pg/ml	1954.99(1002.20,3563.37)	3301.13(1344.63,4512.58)	1727.72(1120.20,3406.03)	1614.08(993.46,3178.76)	1459.03(775.87,4170.69)	**0.029**

**6-minute-walk test**						
6MWD, meters	386.50 ± 87.92	390.70 ± 95.14	387.34 ± 90.85	375.18 ± 80.09	392.87 ± 86.88	0.400

**Echocardiography**						
LVEF, %	62.00(60.00,65.00)	61.00(58.50,65.50)	64.00(61.00,65.50)	61.00(58.00,64.00)	62.00(59.00,65.00)	0.334
E/e’	11.77 ± 3.94	11.67 ± 5.06	10.93 ± 3.65	11.59 ± 3.03	11.47 ± 4.03	0.239
LVMi, g/m^2^	109.70(95.46,126.05)	111.90(99.46,125.96)	114.22(93.00,133.17)	110.37(98.09,126.11)	109.61(95.26,128.81)	0.884
GLS, %	−15.48 ± 2.73	−15.80(−17.80,-12.40)	−15.80(−17.40,-14.00)	−15.50(−17.30,-13.60)	−15.90(−17.80,-12.75)	0.992

BMI, body mass index; WHR, waist-to-hip circumference ratio; BP, blood pressure; MCV, mean corpuscular volume; TSAT, transferrin saturation; CRP, C-reactive protein; eGFR, estimated glomerular filtration rate; PTH, parathyroid hormone; NT-proBNP, N-terminal prohormone brain natriuretic peptide; CK-MB, creatine kinase isoenzymes; iFGF23, fibroblast growth factor-23; 6MWD, 6-minute-walk distance; LVEF, left ventricular ejection fraction; E/e’ ratio, mitral early diastolic inflow velocity (E-wave, m/s) to early diastolic mitral annular velocity (e’, m/s) ratio; LVMi, left ventricular mass index; GLS, global longitudinal strain.

* The P value represents the p for trend in multiple linear regression analysis adjusted for age and gender.

# The P value for One-way ANOVA was >0.05.

### 2.2 Determinants of baseline iFGF23 concentration

In univariable analyses, iFGF23 was associated with PD therapy (β = 0.012, *P* = 0.048), eGFR_CDK-EPI_ (β = −0.007, *P* = 0.010), serum creatinine (β = 0.005, *P* = 0.002), calcium (β = 0.408, *P* = 0.019), and α-Klotho levels (β = −0.004, *P* = 0.001). However, in multivariable linear regression analyses, associations were attenuated and serum α-Klotho (β = −0.042, *P* = 0.013) remained statistically significant and inversely correlated with lgiFGF23 (Table 2). There was no significant association between lgiFGF23 and kidney function, 6MWD, or any echocardiographic parameter including LVMi, E/e’, and GLS.

**Table 2:** Univariable and multivariable association of lgiFGF23 with clinical, laboratory, and echocardiographic variables and exercise capacity in 209 CKD patients.

**Variables**	**Unadjusted**		Adjusted*	
	**β(SE)**	***P* value**	**β(SE)**	***P* value**
Age, yrs	−0.003(0.003)	0.303	−0.003(0.003)	0.280
Male gender	−0.001(0.063)	0.982	0.107(0.134)	0.165
Peritoneal dialysis	0.122(0.061)	**0.048**		
Hemoglobin, g/L	−0.002(0.001)	0.095		
CRP, mg/L	−0.002(0.002)	0.240		
eGFR_CKD-EPI_, ml/min per 1.73 m^2^	−0.007(0.004)	**0.010**		
Urea nitrogen, mg/dL	0.003(0.004)	0.491		
Creatinine, mg/dL	0.005(0.001)	**0.002**		
Serum albumin, g/L	−0.005(0.006)	0.377		
Total cholesterol, mg/dL	−0.002(0.004)	0.640		
Calcium, mmol/L	0.408(0.172)	**0.019**		
Alkaline phosphatase, U/L	−0.001(0.001)	0.529		
PTH, /10 pg/ml	0.001(0.001)	0.757		
NT-proBNP, /10 pg/ml	0.001(0.001)	0.351		
CK-MB, U/L	0.001(0.017)	0.394		
Serum phosphorus, mmol/L	0.089(0.078)	0.253		
α-Klotho, /1000 pg/ml	−0.004(0.001)	**0.001**	−0.042(0.013)	**0.001**
6MWD, /10 m	−0.002(0.004)	0.682		
E/e’	−0.002(0.011)	0.852		
LVMi, g/m^2^	0.001(0.002)	0.572		
GLS, %	0.017(0.014)	0.220		

PTH, parathyroid hormone; NT-proBNP, N-terminal prohormone brain natriuretic peptide; CK-MB, creatine kinase isoenzymes; 6MWD, 6-minute-walk distance; E/e’ ratio, mitral early diastolic inflow velocity (E-wave, m/s) to early diastolic mitral annular velocity (e’, m/s) ratio; LVMi, left ventricular mass index; GLS, global longitudinal strain. SE, standard error. Significant values are shown in bold.

* Take the variables that satisfied P < 0.3 in the univariate correlation analysis as candidates, multivariable regression analysis has been done. Then the stepwise approach was adopted with thresholds of P < 0.05 as inclusion criteria and P > 0.10 as exclusion criteria to keep or remove variables respectively in the multivariable calculation. The adjusted model included age, gender, and α-Klotho. Significant values are shown in bold.

### 2.3 iFGF23 with incident HF and cardiovascular mortality

The univariable Cox regression analyses for the prediction of the primary endpoint are illustrated in Table S1, and each SD increment in lgiFGF23 was significantly associated with the composite endpoint (HR 2.45, 95 %CI 1.31–4.58, *P* = 0.005). In age- and gender-adjusted Cox proportional hazard regression models, each SD increment in lgiFG23 was associated with an estimated 2.3-fold risk of composite endpoints (HR 2.31, 95 %CI 1.24–4.28). In quartile analyses, the risk of composite endpoint was significantly higher in the highest quartile (Q4) compared with the lowest quartile (Q1) of iFGF23 as reference (HR 2.79, 95 %CI 1.37–5.69). HR was only statistically significant in the highest quartile. Similar results were observed in model 2, and model 3 as well as in the fully adjusted model. After adjustments for known risk factors of HF and death, the association between lgiFGF23 and primary endpoint remained independent (HR per SD increase in lgiFGF23 2.33, 95 %CI 1.16–4.68) (Table 3). α-Klotho deficiency was significantly associated with longitudinal outcomes in the univariable analyses but lost statistical significance after multivariable adjustments. The estimates did not attenuate when bone mineral biomarkers including serum calcium, alkaline phosphatase, and PTH as well as echo parameters including LVMi and GLS were added to the fully adjusted model separately (Tables S2 and S3). When taking the lower three quartiles of iFGF23 as a reference, there was a significantly elevated hazard of the composite endpoint with the highest quartile (Q4) of iFGF23 not only in the unadjusted model but also in the fully adjusted model (HR 2.61, 95 %CI 1.49–4.58) (Table S4**)**. The subgroup analyses of 124 PD patients showed that with the increase in iFGF23, the hazard ratios for both the composite endpoint and new-onset HF varied across different adjusted models, suggesting an association between iFGF23 and these outcomes within the PD patient subgroup even after adjusting for multiple relevant factors (Table S5).

**Table 3:** Hazard ratios of the composite endpoint by quartiles of iFGF23 concentration and per SD increase in lgiFGF23 in 209 CKD patients.

	**Hazard Ratio (95 %CI)**				
	**lgiFGF23**	**iFGF23 Q1**	**iFGF23 Q2**	**iFGF23 Q3**	**iFGF23 Q4**
**N, events**	60	11	11	13	25
**Unadjusted**	2.45(1.31,4.58)	Reference	1.01(0.44,2.32)	1.24(0.56,2.77)	2.89(1.41,5.88)
**Model 1**	2.31(1.24,4.28)	Reference	0.98(0.42,2.26)	1.30(0.58,2.91)	2.79(1.37,5.69)
**Model 2**	2.52(1.32,4.78)	Reference	1.02 (0.44,2.39)	1.39(0.61,3.16)	3.33(1.60,6.93)
**Model 3**	2.36(1.18,4.71)	Reference	0.83 (0.35,1.98)	1.33(0.58,3.06)	2.85(1.34,6.04)
**Model 4**	2.33(1.16,4.68)	Reference	0.82(0.34,1.96)	1.33(0.58,3.04)	2.73(1.29,5.77)

Model 1: adjusted for age and gender. Model 2: adjusted for age, gender, smoking history, diabetes mellitus, hypertension and peritoneal dialysis. Model 3: adjusted for age, gender, smoking history, diabetes mellitus, hypertension, peritoneal dialysis, hemoglobin, eGFR_CKD-Epi_, NT-proBNP and α-Klotho. Model 4: adjusted for age, gender, smoking history, diabetes mellitus, hypertension, peritoneal dialysis, hemoglobin, eGFR_CKD-Epi_, NT-proBNP, α-Klotho and E/e’ (fully adjusted model). CI, confidence interval. Q1 refers to the lowest quartile of iFGF23 levels while Q4 refers to the highest quartile.

**Fig. 2:**
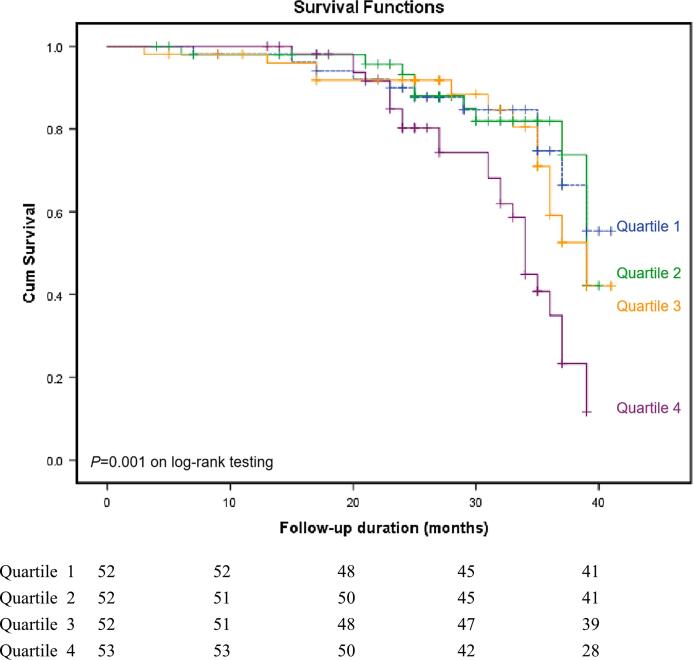
Kaplan-Meier curves for the primary composite endpoint stratified by serum iFGF23 quartiles in 209 CKD patients with preserved EF.

**Fig. 3:**
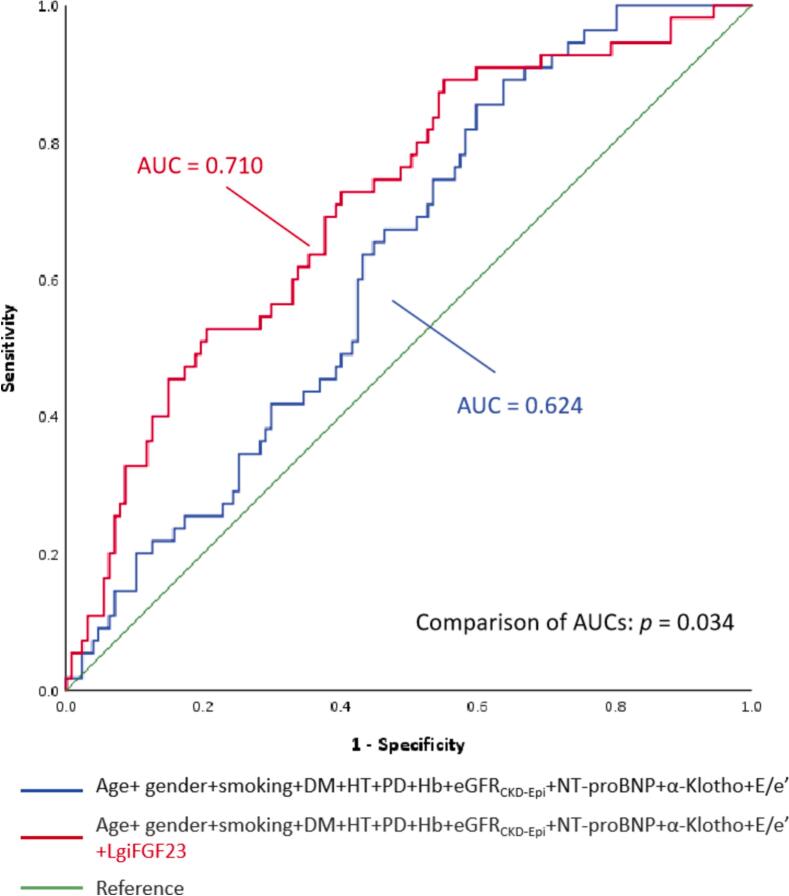
Receiver-Operating Characteristic Curve from adding lgiFGF23 to the conventional and existing risk factors. Incremental value of LgiFGF23. DM **=** diabetes mellitus; HT **=** hypertension; PD **=** peritoneal dialysis; eGFR, estimated glomerular filtration rate; NT-proBNP **=** N-terminal prohormone brain natriuretic peptide; E/e’ **=** mitral early diastolic inflow velocity (E-wave, m/s) to early diastolic mitral annular velocity (e’, m/s) ratio.

Fig. 2 depicts the Kaplan-Meier curves for the primary endpoint (HF and cardiovascular mortality) in the 209 CKD patients stratified by the quartiles of the lgiFGF23 assessment, indicating that patients with the highest iFGF23 quartile (Q4) had the worst prognosis. The 3-year survival ROC analysis showing the incremental value of adding the Q4 level of lgiFGF23 to the traditional cardiovascular risk factors and potential confounders is presented in Fig. 3. The addition of iFGF23 provided incremental value to conventional risk factors.

### 2.4 Sensitivity analyses

**Fig. 4:**
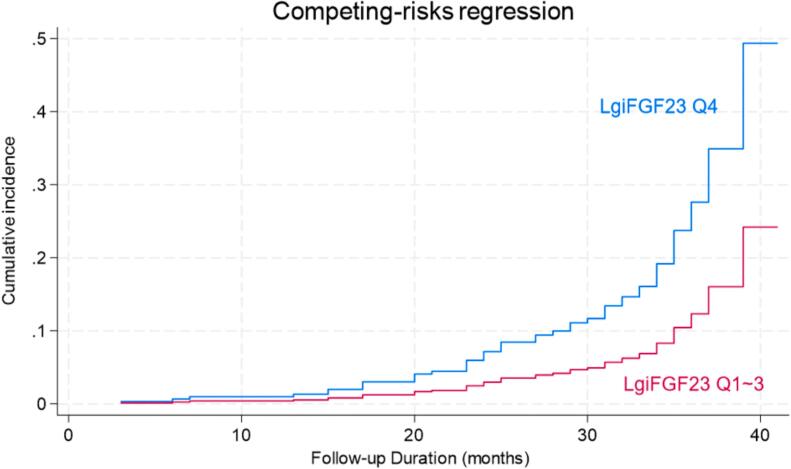
Cumulative incidence estimates of HF and cardiovascular deaths in 209 CKD patients with preserved EF. This analysis stratifies by CKD patients with Q4 level of lgiFGF23 and Q1-3 level of lgiFGF23. Abbreviations as in Table 4**.**

A Competing Risk Cox Regression analysis was used to delineate the confounding effect of non-cardiovascular deaths on the outcomes. The association between the Q4 level of lgiFGF23 and incident HF and cardiovascular death remained after controlling for non-cardiovascular deaths as a competing risk (Table 4). The addition of the Q4 level of lgiFGF23 had incremental predictive value to traditional risk factors and diastolic dysfunction for predicting incident HF and cardiovascular deaths in CKD patients with preserved EF (HR 2.43, 95 %CI 1.44–4.11) (Table 4). The cumulative incidences of new on-set HF and cardiovascular deaths among 209 CKD patients with preserved EF stratified by quartiles (Q1-3 vs Q4) of lgiFGF23 levels are shown in Fig. 4. By the end of the follow-up, cumulative incidences of the primary composite endpoint were about 2-fold higher in patients with the highest quartile (Q4) level of lgiFGF23, compared with lower quartiles (Q1-3) levels of lgiFGF23 (gray test *P* < 0.001).

**Table 4:** Competing risk regression analysis for new-onset HF in 209 CKD patients.

	**Model 1**	**Model 2**	**Model 3**	**Model 4**
Chi-square P for chi-square change	1.38	9.95 *P* = 0.355	15.23 *P* = 0.124	29.36 *P* = **0.002**
	HR (95 %CI) P value	HR (95 %CI) P value	HR (95 %CI) P value	HR (95 %CI), P value
Age ≥ 65yrs	1.16(0.65,2.07) 0.609	1.31(0.61,2.08) 0.695	1.25(0.65,2.39) 0.498	1.18(0.62,2.25) 0.610
Male gender	0.75(0.45,1.24) 0.261	0.97(0.47,1.73) 0.753	0.87(0.44,1.74) 0.705	1.02(0.49,2.22) 0.950
Diabetes Mellitus		1.34(0.60,2.45) 0.590	1.17(0.54, 2.53) 0.680	1.27(0.59,2.73) 0.542
Hypertension		1.30(0.68,2.59) 0.401	1.22(0.62, 2.43) 0.570	1.38(0.65,2.93) 0.402
Ever smoker		1.15(0.66,1.68) 0.809	1.11(0.68, 1.83) 0.669	1.28(0.77,2.12) 0.338
Peritoneal dialysis		1.69(0.85,3.36) 0.131	1.72(0.85,3.48) 0.134	1.66(0.84,3.30) 0.147
Poor Hb		1.96(0.96,3.87) 0.065	1.95(0.97,3.91) 0.057	2.04(1.07,3.87) **0.029**
Poor NT-proBNP		1.19(0.74,2.11) 0.396	1.13(0.66, 1.95) 0.645	1.02(0.60,1.75) 0.918
eGFR < 15 ml/min per 1.73 m^2^		0.71(0.46,2.05) 0.930	0.62(0.25,1.50) 0.286	0.64(0.28,1.50) 0.310
Abnormal E/e’			1.70(0.85,3.37) 0.131	1.56(0.78,3.14) 0.211
FGF23 Q4				2.43(1.44,4.11) **0.001**

FGF23 Q4 showed incremental value over conventional cardiovascular risk factors and diastolic dysfunction for new-onset HF and cardiovascular deaths in CKD patients with preserved EF. Hb = Hemoglobin; NT-proBNP = N-terminal prohormone brain natriuretic peptide; E/e’ ratio = mitral early diastolic inflow velocity (E-wave, m/s) to early diastolic mitral annular velocity (e’, m/s) ratio; FGF23 = fibroblast Growth Factor 23; CI = confidence interval; HR = hazard ratio.

Ever smoker refers to ever-smoked or current smokers. Poor Hb cutoff < 120 g/L for males and 110 g/L for females; Poor NT-proBNP cutoff > 100 pg/ml; Abnormal E/e’ cutoff = 13; FGF23 Q4 > 231.00 ng/ml.

## 3 Discussion

In the present CKD cohort with preserved LVEF, we analyzed whether baseline serum iFGF23 is an independent predictor of new-onset HF and cardiovascular mortality. This study supports two principal conclusions. 1) Serum iFGF23 level elevated even before LVEF deteriorated in CKD stages 3–5. Higher iFGF23 concentration correlates with the drop in serum α-Klotho levels. However, iFGF23 is neither statistically significantly associated with other clinical, laboratory, and echocardiographic parameters, nor with 6WMD when their EF was preserved. 2) Baseline serum iFGF23 concentrations were significantly higher in patients who met the primary endpoint than those without endpoint events. Although previous studies have shown that FGF23 could predict HF in CKD patients, to our knowledge, this is the first study to show that a higher baseline serum iFGF23 level is a strong and independent predictor of HF and cardiac deaths in asymptomatic CKD patients with preserved EF that accounts for non-cardiovascular deaths as a competing risk even after adjusting for potential confounders such as kidney function, traditional cardiovascular risk factors, echocardiographic parameters, and bone mineral biomarkers. Moreover, this association was not even confounded by Klotho deficiency. Overall, these findings confirm prior reports of the association between longitudinal adverse outcomes and baseline iFGF23 levels and extend them by investigating patients with CKD stages 3–5 including patients undergoing PD. In addition, our results suggested that FGF23 shows a predominant role over α-Klotho in the development of cardiac outcomes in CKD. Nevertheless, we did not observe independent associations between iFGF23 and exercise capacity, systemic inflammation, hemoglobin, left ventricular systolic/diastolic dysfunction, myocardial strain, and LVM index when CKD patients had preserved LVEF.

The hormone FGF23, primarily secreted by mature osteoblasts and osteocytes, is essential for regulating the metabolism of minerals and bones [22]. FGF receptors and the co-receptor α-klotho are necessary for the physiological activities of FGF23, which include inhibiting parathyroid hormone (PTH) and promoting phosphaturia. With the deterioration of kidney function, a decrease in α-klotho expression results in a condition of FGF23 resistance. As a result, CKD patients have higher concentrations of FGF23 and PTH and lower levels of α-klotho than health controls [23]. Regarding the relationship between phosphorus levels and FGF23, our study unveiled an interesting aspect. Despite a mean serum phosphorus level of 1.66 ± 0.46 mmol/L, which exceeds the normal range (0.85–1.51 mmol/L), there was no linear correlation between serum phosphorus level and iFGF23. In Univariable Cox regression analysis for the prediction of the primary composite endpoint in patients with CKD, serum phosphorus level also showed no significance. This lack of a straightforward relationship might be attributed to the intricate compensatory and regulatory mechanisms in vivo. For instance, when phosphorus levels deviate from the normal range, multiple feedback loops involving hormones like PTH, vitamin D derivatives, and klotho are activated [24]. These hormones work in concert to maintain mineral balance, and their combined actions could potentially buffer the direct influence of phosphorus on FGF23. Moreover, individual genetic variations play a crucial role in determining the responsiveness of FGF23 to changes in phosphorus levels. Some individuals may possess genetic polymorphisms that modulate the signaling pathways connecting phosphorus sensing and FGF23 secretion, leading to a more complex and less predictable relationship between the two [24].

Consistent with its physiological roles, we observed that iFGF23 significantly correlated with serum α-Klotho levels, calcium levels, and renal function markers including eGFR and creatinine in the univariable analyses. However, no independent association between exercise capacity and iFGF23 was observed in our study. Some studies in other populations have drawn different conclusions. Ghuma et al. [25] and Kanagala et al. [26] suggested that evaluating FGF23 was strongly associated with reduced functional capacity in patients with HF with preserved LVEF (HFpEF). Nevertheless, studies investigating the association of iFGF23 with exercise capacity in CKD patients are limited. Previous studies have shown that in patients with well-controlled mild-to-moderate CKD, their aerobic exercise capacity was preserved over five years as well as cardiovascular and muscular function, despite a minor decrease in GFR. Our data indicate that when the cardiac function of CKD patients is preserved, 6MWD was neither correlated with baseline iFGF23 nor associated with longitudinal adverse outcomes. We suggested that iFGF23 is not correlated with 6MWD and there is no causal link between iFGF23 and physical capacity in this patient group.

In our cohort, 62.7 % are female patients. Despite prior suggestions of gender differences in CKD and HFpEF [27], our study found no significant gender-outcome links, likely due to sample size and disease complexity. The gender imbalance, considering the possible confounding by PD, underlines the need to carefully dissect how the female gender, along with its associated hormonal and comorbidity factors [28], interacts with the pathophysiology of HFpEF in CKD. Future studies with larger, diverse cohorts are needed to understand gender's role and interactions with FGF23, aiming to help female CKD patients at risk of HFpEF. In addition, our subgroup analysis suggests that after adjusting for dialysis duration, the predictive value of iFGF23 for incident HF and cardiovascular mortality persists in PD patients. However, further studies are needed to explore the potential modifying effects of dialysis-related factors on the FGF23-cardiovascular disease relationship.

Among patients with CKD, left ventricular hypertrophy (LVH) is one of the most frequent conditions with cardiovascular complications [6]. Animal studies and vitro studies confirmed that FGF23 could promote hypertrophy in cardiomyocytes, which is mediated by FGF23 binding to FGFR4 in a klotho-independent action [29], [30]. In cardiac cells, FGF23 could bind to FGFR4 stimulating phospholipase Cγ (PLCγ) phosphorylation and downstream initiating of the calcineurin/NFAT signaling pathway, which, in turn, promotes cardiac remodeling [6], [31]. In support of direct evidence connecting FGF23-FGFR4 binding to LVH development, Han et al [32] found that cardiomyocyte-specific *Fgfr4*-knockout mice were resistant to FGF23-induced cardiac hypertrophy by repeated injection of recombinant FGF23. Contrariwise, mice with a single amino acid substitution-induced constitutive gain-of-function mutation in *Fgfr4* develop LVH without kidney damage or increased FGF23 levels [30]. Overall, in mice models of CKD, selective inhibition of FGFR4 or calcineurin or global inhibition of all FGFRs attenuates pathological cardiac remodeling. Similarly, in mice with CKD, pharmacological or genetic interventions that lower serum FGF23 levels or inhibit all FGFRs can attenuate LVH without affecting blood pressure or kidney function [6]. However, these preclinical findings are only tangentially corroborated by clinical data due to the inherent limits of human research. Outcomes across many observational studies revealed that concentric LVH was significantly associated with higher FGF23 levels in individuals with non-dialysis CKD [33], [34], [35] and patients receiving hemodialysis (HD) [36], [37]. Meanwhile, not all studies support the link between FGF23 and LVH. In a study that enrolled 83 children with non-dialysis CKD stages 3–5, researchers failed to establish a significant correlation between FGF23 and LVH [38]. Our finding also suggested that in adult patients with stages 3–5 CKD including patients receiving PD, serum iFGF23 and LVH were not correlated. Likewise, using gold-standard cardiac magnetic resonance (CMR) imaging, Kanagala et al. [26] failed to find any association between LV mass indices and FGF23 indices in HFpEF patients. Of note, the methodologies, the stages of CKD, the characteristics of participants enrolled, the imaging techniques, and the covariates in the multivariate regression models were all likely to contribute to the variation of these results. Beyond this, controversy exists concerning standardizing the most frequent four commercially available FGF23 assays in clinical use, with incorporated measures suggesting different conclusions. For instance, in the studies mentioned above, Gutiérrez et al [34] measured cFGF23 serum levels in 162 CKD patients, whereas Sinha et al. [38] measured iFGF23 in 83 children. There is proof that compared to cFGF23, iFGF23 may more accurately reflect biological importance, and the C-terminal fragments could counter-regulate the active full-length version [39], [40].

Previous research has revealed associations between FGF23 and the new-onset HF, in the general population [15], [41], [42], [43], patients with non-dialyzed CKD [13], [43], [44], and patients undergoing HD [45]. Our results were consistent with previous literature supporting the association of raised iFGF23 with HF and cardiovascular death and, importantly, extended the conclusion in patients with CKD stages 3–5 including PD patients. Moreover, we observed that FGF23 elevated even before LVEF deteriorated in CKD patients. This recognition of elevation is important because it provides the rationale for initiating cardioprotective strategies in this population. Conventionally, in clinical practice, LVEF is one of the most important echocardiographic parameters that influences treatment decisions despite its low sensitivity and specificity in predicting cardiac death. The elevation of FGF23 may occur before any other echocardiographic marker of cardiac dysfunction and mirror the raised cardiovascular risks which allows the classification of asymptomatic CKD patients who are at risk of symptomatic and advanced HF and cardiac death. However, when we ordered iFGF23 concentration in quartiles, there was no trend in HRs across the top to the bottom thirds. The absence of a dose–response relationship suggests a threshold effect, where a higher concentration of iFGF23 is more detrimental than intermediate and lower doses. Moreover, increased iFGF23 level was found to offer incremental prognostic information over clinical and demographic risk factors, biological biomarkers, and other echo parameters in the prediction of symptomatic HF and cardiac death in patients with CKD stage 3–5 and preserved LVEF.

The distal tubules of the kidney are the primary location where klotho is produced. In CKD, a reduction in functional nephrons and suppression of *Klotho* expression can lead to a deficiency in Klotho. In keeping with previous research [3], [29], we found that the reduced circulating level of klotho was linearly correlated with increased FGF23 concentration. Nevertheless, we failed to detect the correlation between klotho levels and endpoint events, which is similar to recently published research data from the CRIC study [46]. Importantly, we also explored the association between the ratio of circulating FGF23 to Klotho with endpoints but failed to find any evidence. Hence, based on our current knowledge of the FGF23/Klotho axis interacting with components of stress response such as the renin-angiotensin system (RAS), and reactive oxygen species (ROS), we could speculate that FGF23 may act as a stress hormone and initiate the stress response, which in turn downregulates serum Klotho and ultimately amplifies the stress reaction. An *in vivo* investigation indicated that the hypertrophy of the heart and cardiomyocytes caused by FGF23 is reversible [47]. This illustrates that the hypertrophy caused by FGF23 can be reversed by increasing the contractile force. After the stressor (FGF23) is removed, the stress response attenuates leading to a reduction in cardiac hypertrophy. Recent findings imply that soluble Klotho may lessen some of these effects through various potential processes that are not yet fully comprehended [6]. Our findings highlight the possibility that the secretion of iFGF23 increases is more important in terms of pathophysiology primary than Klotho deficiency as it may respond to the increased phosphate level and renal impairment progression and then trigger the stress response. Elucidating the interaction between stress response and FGF23/Klotho axis in cardiomyocytes may offer a new perspective for future research.

Our study indicates that baseline iFGF23 levels independently predict incident HF and cardiovascular mortality in CKD patients with preserved LVEF, suggesting its utility as a biomarker for risk assessment. Clinically, patients with elevated iFGF23 might need closer surveillance. Regarding treatment, current evidence [3] indicates that certain interventions can impact FGF23. For example, dietary phosphate management could be considered due to phosphate's influence on FGF23. Although no specific iFGF23-targeted therapies exist for CKD patients currently, our results support further research. For early interventions, lifestyle changes such as dietary phosphate restriction and regular exercise could be explored. Exercise has been associated with an increase in α-Klotho expression [3]. In our study, no direct exercise-iFGF23 association was found, but future studies may clarify. In summary, iFGF23 holds clinical potential, and future efforts should focus on developing targeted therapies and early interventions based on it to improve patients' cardiovascular prognosis.

Our study has some limitations. iFGF23 is an individual biomarker involved in intricate processes related to bone and phosphate regulation. These pathways contain other toxic factors that could hasten the onset of HF and cause death. We could not determine whether the iFGF23 level is a surrogate marker of other toxic factors in this study. Thus, further research is needed to explore these possibilities. Furthermore, we acknowledge that our study has a relatively small sample size. Although we observed significant associations between baseline serum iFGF23 levels and incident HF and cardiovascular mortality, a larger sample could provide more precise estimates and enhance the generalizability of our findings. The limited sample size might have precluded us from detecting more subtle relationships or dose–response patterns. Future studies with a larger number of participants are warranted to further validate and expand upon our results. Another limitation pertains to the lack of data on medications that could potentially influence iFGF23 levels. Medications such as phosphate binders, vitamin D analogs, and diuretics play roles in the complex pathophysiology of CKD and associated disorders. However, due to various reasons including incomplete collection or inaccurate patient recall, we were unable to incorporate this information into our analyses. This omission may confound and obscure the iFGF23-outcome relation. Future studies must prioritize comprehensive medication data collection to address this. Additionally, the study conducted at only one center is a notable potential limitation.

In conclusion, we observed that serum iFGF23 levels were elevated even before LVEF declined in patients with CKD stages 3–5, including those on PD. A higher serum iFGF23 level is independently associated with incident HF and cardiovascular mortality over a 3-year follow-up, and the association is not confounded by Klotho deficiency. An increased iFGF23 concentration adds incremental prognostic value beyond conventional clinical and laboratory cardiovascular risk factors and other echocardiographic parameters in this population.

## Sources of funding

This research was supported by the National Natural Science Foundation of China for Young Scientists (grant number:82200833); the Fund for Scientific Research of Anhui Medical University (grant number:2019xkj011); the Fund for Postdoctoral Research of the First Affiliated Hospital of Anhui Medical University (grant number:1458).

## CRediT authorship contribution statement

**Ying Wang:** Writing – original draft, Visualization, Resources, Project administration, Methodology, Investigation, Funding acquisition, Formal analysis, Data curation, Conceptualization. **Dingxin Zhang:** Resources, Methodology, Data curation. **Runzhe Zhou:** Methodology, Investigation, Data curation. **Xiangjie Yang:** Project administration, Methodology, Investigation, Data curation. **Xiaoxia Wang:** Project administration, Methodology, Investigation. **Yuxin Jiang:** Project administration, Methodology, Investigation. **Xinyuan Zhou:** Project administration, Methodology, Investigation. **Dashan Li:** Validation, Investigation, Formal analysis. **Jin Zhang:** Supervision, Project administration, Investigation. **Yonggui Wu:** Writing – review & editing, Supervision.

## Declaration of competing interest

The authors declare that they have no known competing financial interests or personal relationships that could have appeared to influence the work reported in this paper.

## References

[1] Dilsizian V., Gewirtz H., Marwick T.H., Kwong R.Y., Raggi P., Al-Mallah M.H., Herzog C.A. (2021). Cardiac imaging for coronary heart disease risk stratification in chronic kidney disease. J. Am. Coll. Cardiol. Img..

[2] Li X., Lindholm B. (2022). Cardiovascular risk prediction in chronic kidney disease. Am. J. Nephrol..

[3] Law J.P., Price A.M., Pickup L., Radhakrishnan A., Weston C., Jones A.M., McGettrick H.M., Chua W., Steeds R.P., Fabritz L. (2020). Clinical potential of targeting fibroblast growth factor-23 and alphaKlotho in the treatment of uremic cardiomyopathy. J. Am. Heart Assoc..

[4] Kovesdy C.P., Anderson J.E. (2007). Reverse epidemiology in patients with chronic kidney disease who are not yet on dialysis. Semin. Dial..

[5] Kalantar-Zadeh K., Block G., Humphreys M.H., Kopple J.D. (2003). Reverse epidemiology of cardiovascular risk factors in maintenance dialysis patients. Kidney Int..

[6] Edmonston D., Grabner A., Wolf M. (2023). FGF23 and klotho at the intersection of kidney and cardiovascular disease. Nat. Rev. Cardiol..

[7] Memmos E., Papagianni A. (2021). New insights into the role of FGF-23 and klotho in cardiovascular disease in chronic kidney disease patients. Curr. Vasc. Pharmacol..

[8] Vervloet M. (2019). Renal and extrarenal effects of fibroblast growth factor 23. Nat. Rev. Nephrol..

[9] Kato K., Jeanneau C., Tarp M.A., Benet-Pagès A., Lorenz-Depiereux B., Bennett E.P., Mandel U., Strom T.M., Clausen H. (2006). Polypeptide GalNAc-transferase T3 and familial tumoral calcinosis. Secretion of fibroblast growth factor 23 requires O-glycosylation. J. Biol. Chem..

[10] Zhang X., Guo K., Xia F., Zhao X., Huang Z., Niu J. (2018). FGF23(C-tail) improves diabetic nephropathy by attenuating renal fibrosis and inflammation. BMC Biotech..

[11] Roy C., Lejeune S., Slimani A., de Meester C., Ahn As S.A., Rousseau M.F., Mihaela A., Ginion A., Ferracin B., Pasquet A. (2020). Fibroblast growth factor 23: a biomarker of fibrosis and prognosis in heart failure with preserved ejection fraction. ESC Heart Fail..

[12] Kurosu H., Ogawa Y., Miyoshi M., Yamamoto M., Nandi A., Rosenblatt K.P., Baum M.G., Schiavi S., Hu M.C., Moe O.W., Kuro-o M. (2006). Regulation of fibroblast growth factor-23 signaling by klotho. J. Biol. Chem..

[13] Leidner A.S., Cai X., Zelnick L.R., Lee J., Bansal N., Pasch A., Kansal M., Chen J., Anderson A.H., Sondheimer J.H. (2023). Fibroblast Growth factor 23 and risk of heart failure subtype: the CRIC (Chronic Renal Insufficiency Cohort) study. Kidney Med..

[14] Marthi A., Donovan K., Haynes R., Wheeler D.C., Baigent C., Rooney C.M., Landray M.J., Moe S.M., Yang J., Holland L. (2018). Fibroblast growth factor-23 and risks of cardiovascular and noncardiovascular diseases: a meta-analysis. J. Am. Soc. Nephrol..

[15] Liu M., Xia P., Tan Z., Song T., Mei K., Wang J., Ma J., Jiang Y., Zhang J., Zhao Y. (2022). Fibroblast growth factor-23 and the risk of cardiovascular diseases and mortality in the general population: a systematic review and dose-response meta-analysis. Front. Cardiovasc. Med..

[16] Levey A.S., Eckardt K.U., Dorman N.M., Christiansen S.L., Hoorn E.J., Ingelfinger J.R., Inker L.A., Levin A., Mehrotra R., Palevsky P.M. (2020). Nomenclature for kidney function and disease: report of a kidney disease: improving global outcomes (KDIGO) consensus conference. Kidney Int..

[17] Ho K. (1993). The epidemiology of heart failure: the Framingham Study. J. Am. Coll. Cardiol..

[18] Inker L.A., Schmid C.H., Tighiouart H., Eckfeldt J.H., Feldman H.I., Greene T., Kusek J.W., Manzi J., Van Lente F., Zhang Y.L. (2012). Estimating glomerular filtration rate from serum creatinine and cystatin C. N. Engl. J. Med..

[19] ATS statement: guidelines for the six-minute walk test, Am. J. Respir. Crit. Care Med. 2002 (166) 111–117, doi: 10.1164/ajrccm.166.1.at1102. 10.1164/ajrccm.166.1.at1102 12091180

[20] Lang R.M., Badano L.P., Mor-Avi V., Afilalo J., Armstrong A., Ernande L., Flachskampf F.A., Foster E., Goldstein S.A., Kuznetsova T. (2015). Recommendations for cardiac chamber quantification by echocardiography in adults: an update from the American Society of Echocardiography and the European Association of Cardiovascular Imaging. Eur. Heart J. – Cardiovasc. Imaging..

[21] Nagueh S.F., Smiseth O.A., Appleton C.P., Byrd B.F., Dokainish H., Edvardsen T., Flachskampf F.A., Gillebert T.C., Klein A.L., Lancellotti P. (2016). Recommendations for the evaluation of left ventricular diastolic function by echocardiography: an update from the American Society of Echocardiography and the European Association of Cardiovascular Imaging. J. Am. Soc. Echocardiogr..

[22] Bishop N., Burton J., Graham-Brown M., Stensel D., Viana J., Watson E. (2023). Exercise and chronic kidney disease: potential mechanisms underlying the physiological benefits. Nat. Rev. Nephrol..

[23] Cannata-Andia J.B., Martin-Carro B., Martin-Virgala J., Rodriguez-Carrio J., Bande-Fernandez J.J., Alonso-Montes C., Carrillo-Lopez N. (2021). Chronic kidney disease-mineral and bone disorders: pathogenesis and management. Calcif. Tissue Int..

[24] Portales-Castillo I., Simic P. (2022). PTH, FGF-23, Klotho and Vitamin D as regulators of calcium and phosphorus: genetics, epigenetics and beyond. Front. Endocrinol. (Lausanne)..

[25] Ghuman J., Cai X., Patel R.B., Khan S.S., Hecktman J., Redfield M.M., Lewis G., Shah S.J., Wolf M., Isakova T., Mehta R. (2021). Fibroblast growth factor 23 and exercise capacity in heart failure with preserved ejection fraction. J. Card. Fail..

[26] Kanagala P., Arnold J.R., Khan J.N., Singh A., Gulsin G.S., Eltayeb M., Gupta P., Squire I.B., McCann G.P., Ng L.L. (2020). Fibroblast-growth-factor-23 in heart failure with preserved ejection fraction: relation to exercise capacity and outcomes. ESC Heart Fail..

[27] Lofman I., Szummer K., Dahlstrom U., Jernberg T., Lund L.H. (2017). Associations with and prognostic impact of chronic kidney disease in heart failure with preserved, mid-range, and reduced ejection fraction. Eur. J. Heart Fail..

[28] Tippen S.P., Noonan M.L., Ni P., Metzger C.E., Swallow E.A., Sacks S.A., Chen N.X., Thompson W.R., Prideaux M., Atkins G.J. (2021). Age and sex effects on FGF23-mediated response to mild phosphate challenge. Bone.

[29] Bao J.F., Hu P.P., She Q.Y., Li A. (2020). A land of controversy: fibroblast growth factor-23 and uremic cardiac hypertrophy. J. Am. Soc. Nephrol..

[30] Grabner A., Amaral A.P., Schramm K., Singh S., Sloan A., Yanucil C., Li J., Shehadeh L.A., Hare J.M., David V. (2015). Activation of cardiac fibroblast growth factor receptor 4 causes left ventricular hypertrophy. Cell Metab..

[31] Wilkins B.J., Dai Y.S., Bueno O.F., Parsons S.A., Xu J., Plank D.M., Jones F., Kimball T.R., Molkentin J.D. (2004). Calcineurin/NFAT coupling participates in pathological, but not physiological, cardiac hypertrophy. Circ. Res..

[32] Han X., Cai C., Xiao Z., Quarles L.D. (2020). FGF23 induced left ventricular hypertrophy mediated by FGFR4 signaling in the myocardium is attenuated by soluble Klotho in mice. J. Mol. Cell. Cardiol..

[33] Faul C., Amaral A.P., Oskouei B., Hu M.C., Sloan A., Isakova T., Gutiérrez O.M., Aguillon-Prada R., Lincoln J., Hare J.M. (2011). FGF23 induces left ventricular hypertrophy. J. Clin. Invest..

[34] Gutiérrez O.M., Januzzi J.L., Isakova T., Laliberte K., Smith K., Collerone G., Sarwar A., Hoffmann U., Coglianese E., Christenson R. (2009). Fibroblast growth factor 23 and left ventricular hypertrophy in chronic kidney disease. Circulation.

[35] Mitsnefes M.M., Betoko A., Schneider M.F., Salusky I.B., Wolf M.S., Jüppner H., Warady B.A., Furth S.L., Portale A.A. (2018). FGF23 and left ventricular hypertrophy in children with CKD. Clin. J. Am. Soc. Nephrol.: CJASN.

[36] Negishi K., Kobayashi M., Ochiai I., Yamazaki Y., Hasegawa H., Yamashita T., Shimizu T., Kasama S., Kurabayashi M. (2010). Association between fibroblast growth factor 23 and left ventricular hypertrophy in maintenance hemodialysis patients. Comparison with B-type natriuretic peptide and cardiac troponin T. Circ. J..

[37] Seeherunvong W., Abitbol C.L., Chandar J., Rusconi P., Zilleruelo G.E., Freundlich M. (2012). Fibroblast growth factor 23 and left ventricular hypertrophy in children on dialysis. Pediatr. Nephrol..

[38] Sinha M.D., Turner C., Booth C.J., Waller S., Rasmussen P., Goldsmith D.J., Simpson J.M. (2015). Relationship of FGF23 to indexed left ventricular mass in children with non-dialysis stages of chronic kidney disease. Pediatr. Nephrol..

[39] Goetz R., Nakada Y., Hu M.C., Kurosu H., Wang L., Nakatani T., Shi M., Eliseenkova A.V., Razzaque M.S., Moe O.W. (2010). Isolated C-terminal tail of FGF23 alleviates hypophosphatemia by inhibiting FGF23-FGFR-Klotho complex formation. PNAS.

[40] Grund A., Sinha M.D., Haffner D., Leifheit-Nestler M. (2021). Fibroblast growth factor 23 and left ventricular hypertrophy in chronic kidney disease-a pediatric perspective. Front. Pediatr..

[41] Kestenbaum B., Sachs M.C., Hoofnagle A.N., Siscovick D.S., Ix J.H., Robinson-Cohen C., Lima J.A., Polak J.F., Blondon M., Ruzinski J. (2014). Fibroblast growth factor-23 and cardiovascular disease in the general population: the Multi-Ethnic Study of Atherosclerosis. Circ. Heart Fail..

[42] Garimella P.S., Ix J.H., Katz R., Chonchol M.B., Kestenbaum B.R., de Boer I.H., Siscovick D.S., Shastri S., Hiramoto J.S., Shlipak M.G., Sarnak M.J. (2014). Fibroblast growth factor 23, the ankle-brachial index, and incident peripheral artery disease in the Cardiovascular Health Study. Atherosclerosis.

[43] Scialla J.J., Xie H., Rahman M., Anderson A.H., Isakova T., Ojo A., Zhang X., Nessel L., Hamano T., Grunwald J.E. (2014). Fibroblast growth factor-23 and cardiovascular events in CKD. J. Am. Soc. Nephrol..

[44] Seiler S., Rogacev K.S., Roth H.J., Shafein P., Emrich I., Neuhaus S., Floege J., Fliser D., Heine G.H. (2014). Associations of FGF-23 and sKlotho with cardiovascular outcomes among patients with CKD stages 2–4. Clin. J. Am. Soc. Nephrol.: CJASN..

[45] Kang M., Chen J., Liu L., Xue C., Tang X., Lv J., Fu L., Mei C., Mao Z., Liu Y., Dai B. (2022). In-center nocturnal hemodialysis reduced the circulating FGF23, left ventricular hypertrophy, and all-cause mortality: a retrospective cohort study. Front. Med. (Lausanne)..

[46] Edmonston D., Fuchs M.A.A., Burke E.J., Isakova T., Wolf M., Appel L.J., Chen J., Cohen D.L., Feldman H.I., Go A.S. (2024). Klotho and clinical outcomes in CKD: findings from the chronic renal insufficiency cohort (CRIC) study. Am. J. Kidney Dis..

[47] Touchberry C.D., Green T.M., Tchikrizov V., Mannix J.E., Mao T.F., Carney B.W., Girgis M., Vincent R.J., Wetmore L.A., Dawn B. (2013). FGF23 is a novel regulator of intracellular calcium and cardiac contractility in addition to cardiac hypertrophy. Am. J. Phys. Endocrinol. Metab..

