# Supplementary material for: Baseline fibroblast growth factor 23 predicts incident heart failure and cardiovascular mortality in patients with chronic kidney disease: A 3-year follow-up study
Source: Int J Cardiol Heart Vasc. 2024 Dec 23;56:101587. doi: 10.1016/j.ijcha.2024.101587 (PMC11728072; doi:10.1016/j.ijcha.2024.101587)
Supplement: Supplementary Data 1 [file mmc1.docx]

**Supplement Table S1.** Univariable Cox regression analysis for the prediction of the primary composite endpoint in patients with CKD.

| **Variables** | **Unadjusted** | |
| --- | --- | --- |
|  | **HR (95%CI)** | ***P* value** |
| **Demographic and clinical characteristics** | | |
| Age, yrs | 1.02(0.99,1.04) | 0.180 |
| Male gender | 0.75(0.45,1.25) | 0.264 |
| BMI, kg/m^2^ | 1.03 (0.96,1.11) | 0.387 |
| WHR | 2.43(0.19,31.35) | 0.496 |
| Current smoking | 0.98(0.39,2.48) | 0.970 |
| Heart rate, beats/min | 0.99(0.98,1.01) | 0.510 |
| Systolic BP, mm Hg | 1.00(0.99,1.02) | 0.641 |
| Diastolic BP, mm Hg | 0.99(0.97,1.011) | 0.353 |
| Hypertension | 1.47(0.76,2.83) | 0.251 |
| Diabetes | 1.48(0.79,2.73) | 0.215 |
| Peritoneal dialysis | 1.31(0.86,2.24) | 0.331 |
| **Biochemical parameters** | | |
| Hemoglobin, g/L | 0.98(0.97,0.99) | **0.001** |
| Iron, μmol/L | 0.94(0.89,1.01) | 0.079 |
| TSAT, % | 0.98(0.96,1.01) | 0.174 |
| Ferritin, μg/L | 1.01(0.99,1.02) | 0.445 |
| CRP, mg/L | 1.01(0.99,1.03) | 0.172 |
| Urea nitrogen, mg/dL | 1.01(0.99,1.02) | 0.174 |
| Creantinie, mg/dL | 1.03(0.97,1.08) | 0.337 |
| eGFR_CKD-EPI,_ ml/min per 1.73m^2^ | 0.98(0.96,1.01) | 0.133 |
| Serum albumin, g/L | 0.96(0.93,1.01) | 0.136 |
| Total cholesterol, mg/dL | 0.99(0.96,1.03) | 0.681 |
| Calcium, mmol/L | 0.74(0.25,2.20) | 0.588 |
| Alkaline phosphatase, U/L | 1.01(0.98,1.02) | 0.230 |
| PTH, pg/ml | 1.00(0.99,1.01) | 0.242 |
| NT-proBNP, pg/ml | 1.00(0.99,1.01) | 0.639 |
| CK-MB, /10 U/L | 1.04(0.96,1.13) | 0.351 |
| Serum phosphorus, mmol/L | 1.13(0.67,1.90) | 0.651 |
| α-Klotho, /1000 pg/ml | 1.01(1.00,1.21) | **0.050** |
| iFGF23, /100 ng/ml | 1.13(1.04,1.23) | **0.005** |
| lgiFGF23 | 2.39(1.27,4.51) | **0.007** |
| **6-minute-walk test** | | |
| 6MWD, /10 meters | 0.98(0.95,1.01) | 0.124 |
| **Echocardiography** | | |
| LVEF, % | 0.95(0.89,1.01) | 0.089 |
| E/e’ | 1.12(1.03,1.23) | **0.011** |
| LVMi, g/m^2^ | 1.01(0.99,1.02) | 0.134 |
| GLS, % | 1.08(0.95,1.21) | 0.234 |

HR, hazard ratio. CI, confidence interval; Significant values are shown in bold. BMI, body mass index; WHR, waist-to-hip circumference ratio; BP, blood pressure; TSAT, transferrin saturation; CRP, C-reactive protein; eGFR, estimated glomerular filtration rate; PTH, parathyroid hormone; NT-proBNP, N-terminal prohormone brain natriuretic peptide; CK-MB, creatine kinase isoenzymes; LgiFGF23, log10-transformed intact fibroblast growth factor-23. 6MWD, 6-minute-walk distance; LVEF, left ventricular ejection fraction; E/e’ ratio, mitral early diastolic inflow velocity (E-wave, m/s) to early diastolic mitral annular velocity (e’, m/s) ratio; LVMi, left ventricular mass index; GLS, global longitudinal strain; Significant values are shown in bold.

**Supplement Table S2.** Hazard ratios of heart failure and cardiovascular death per SD increase in lgiFGF23 of the fully adjusted model supplemented with bone mineral biomarkers in 209 CKD patients with preserved EF.

|  | **HR (95%CI)** | ***P* value** |
| --- | --- | --- |
| **Model 4^*^** | | |
| **+ Serum calcium** | 2.35(1.19,4.63) | **0.014** |
| **+** **Alkaline phosphatase** | 2.49(1.26,4.92) | **0.009** |
| **+ PTH** | 2.51(1.26,4.99) | **0.009** |

PTH, parathyroid hormone; HR, hazard ratio. CI, confidence interval.

^*^Model 4 was adjusted for age, gender, smoking history, peritoneal dialysis, diabetes mellitus, hypertension, hemoglobin, eGFR_CKD-Epi,_ NT-proBNP, α-Klotho, and E/e’ (fully adjusted). Significant values are shown in bold.

**Supplement Table S3.** Hazard ratios of heart failure and cardiovascular death per SD increase in lgiFGF23 of the fully adjusted model supplemented with echocardiographic biomarkers in 209 CKD patients with preserved EF.

|  | **HR (95%CI)** | ***P* value** |
| --- | --- | --- |
| **Model 4^*^** | | |
| **+ LVM index** | 2.38(1.22,4.66) | **0.011** |
| **+ GLS** | 2.29(1.17,4.46) | **0.015** |

PTH, parathyroid hormone; HR, hazard ratio. CI, confidence interval.

^*^Model 4 was adjusted for age, gender, smoking history, peritoneal dialysis, diabetes mellitus, hypertension, hemoglobin, eGFR_CKD-Epi,_ NT-proBNP, α-Klotho, and E/e’ (fully adjusted). Significant values are shown in bold.

**Supplement Table S4**. Association of baseline serum iFGF23 levels in quartiles with the primary composite endpoint in 209 CKD patients with preserved EF.

| **Variables** | **Unadjusted** | | **Adjusted^*^** | |
| --- | --- | --- | --- | --- |
|  | **HR (95%CI)** | ***P* value** | **HR (95%CI)** | ***P* value** |
| **iFGF23** | | | | |
| **iFGF23 Quartiles 1-3** | Reference |  | Reference |  |
| **iFGF23 Quartiles 4** | 2.67(1.59,4.49) | **<0.001** | 2.61(1.49,4.58) | **0.001** |

^*^The adjusted model was adjusted for age, gender, smoking history, peritoneal dialysis, diabetes mellitus, hypertension, hemoglobin, eGFR_CKD-Epi_, NT-proBNP, α-Klotho, and E/e’ (fully adjusted).

iFGF23, intact fibroblast growth factor-23; HR, hazard ratio. CI, confidential interval; Significant values are shown in bold.

**Table S5.** Hazard ratios of the composite endpoint by per SD increase in lgiFGF23 and competing risk regression analysis for new-onset HF by FGF23 Q4 in 124 PD patients.

|  | **Hazard Ratio (95%CI)** | |
| --- | --- | --- |
|  | **Composite endpoint** | **New-onset HF** |
| **N, events** | 37 | 33 |
| **Unadjusted** | 2.44(1.10,5.40) | 2.53(1.37,4.67) |
| **Model 1** | 2.42(1.12,5.22) | 2.47(1.32,4.61) |
| **Model 2** | 3.01(1.36,7.07) | 3.14(1.62,6.06) |
| **Model 3** | 2.74(1.01,7.45) | 3.22(1.63,6.35) |
| **Model 4** | 3.49(1.20,10.11) | 3.03(1.50,6.13) |

Model 1: adjusted for age and gender. Model 2: adjusted for age, gender, smoking history, diabetes mellitus, hypertension, and peritoneal dialysis duration. Model 3: adjusted for age, gender, smoking history, diabetes mellitus, hypertension, peritoneal dialysis duration, hemoglobin, eGFR_CKD-Epi_, NT-proBNP and α-Klotho. Model 4: adjusted for age, gender, smoking history, diabetes mellitus, hypertension, peritoneal dialysis duration, hemoglobin, eGFR_CKD-Epi_, NT-proBNP, α-Klotho and E/e’ (fully adjusted model). CI, confidence interval.
